# Serum sonic hedgehog (SHH) and interleukin-(IL-6) as dual prognostic biomarkers in progressive metastatic breast cancer

**DOI:** 10.1038/s41598-017-01268-4

**Published:** 2017-05-11

**Authors:** A. S. Noman, M. Uddin, A. A. Chowdhury, M. J. Nayeem, Z. Raihan, M. I. Rashid, A. K. Azad, M. L. Rahman, D. Barua, A. Sultana, A. Shirin, J. Ferdous, R. R. Parag, S. M. Rahman, M. R. Mahmud, C. S. Jerin, N. Jahan, A. Siddiqua, T. Ara, E. B. Sabur, S. S. Alam, S. Baidya, S. Akther, M. Z. Rahman, T. Banu, A. K. Murugan, S. Sabri, S. M. S. Islam, B. Karakas, A. Aboussekhra, H. Yeger, W. A. Farhat, S. S. Islam

**Affiliations:** 1 Department of Pathology, McGill University and Cancer Research Program, The Research Institute of the McGill University Health Centre, Montreal, Quebec, Canada; 2 The Centre for Applied Genomics, The Hospital for Sick Children, Toronto, ON Canada; 3 Mohammed Bin Rashid University of Medicine and Health Sciences, College of Medicine, Dubai, UAE; 4 Department of Radiotherapy, Chittagong Medical College Hospital, Chittagong, Bangladesh; 5 Department of Biochemistry and Molecular Biology, University of Chittagong, Chittagong, Bangladesh; 6 Department of General Surgery, Chittagong Medical College Hospital, Chittagong, Bangladesh; 7 Department of Genetic Engineering and Biotechnology, University of Chittagong, Chittagong, Bangladesh; 8 Department of Pathology, Chittagong Medical College Hospital, Chittagong, Bangladesh; 9 Department of Pediatric Surgery, Chittagong Medical College Hospital, Chittagong, Bangladesh; 10 Division of Molecular Oncology, King Faisal Specialist Hospital and Research Centre, Riyadh, Saudi Arabia; 11 Department of Oncology, McGill University and Cancer Research Program, The Research Institute of the McGill University Health Centre, Montreal, Quebec, Canada; 12 Department of Pediatric Cardiology, Royal Hospital, Muscat, Oman; 13 Developmental and Stem Cell Biology Research Institute, The Hospital for Sick Children, Toronto, ON Canada; 14 Department of Biochemistry and Molecular Biology, University of Chittagong, Chittagong, Bangladesh; 15 Division of Molecular Oncology, King Faisal Specialist Hospital and Research Centre, Riyadh, Saudi Arabia

## Abstract

Serum from one hundred and ten breast cancer patients and thirty healthy female volunteers, were prospectively collected and evaluated for serum levels of Shh and IL-6 using human Shh and IL-6 specific enzyme-linked immunoassays. All patients were regularly monitored for event free survival (EFS) and overall survival (OS). Overall outcome analysis was based on serum Shh and IL-6 levels. In patients with progressive metastatic BC, both serum Shh and IL-6 concentrations were elevated in 44% (29 of 65) and 63% (41 of 65) of patients, respectively, at a statistically significant level [Shh (p = 0.0001) and IL-6 (p = 0.0001)] compared to the low levels in healthy volunteers. Serum levels tended to increase with metastatic progression and lymph node positivity. High serum Shh and IL-6 levels were associated with poor EFS and OS opposite to the negative or lower levels in serum Shh and IL-6. The elevated levels of both serum Shh and IL-6 were mainly observed in BC patients who had a significantly higher risk of early recurrence and bone metastasis, and associated with a worse survival for patients with progressive metastatic BC. Further studies are warranted for validating these biomarkers as prognostic tools in a larger patient cohort and in a longer follow-up study.

## Introduction

Traditional prognostic factors for breast cancer (BC) patients are largely based on careful histological analysis of tumor size, tumor grade, lymph node metastasis and tumor subtypes. Many molecular markers such as estrogen receptor (ER), progesterone receptor (PR) and human epidermal growth factor receptor 2 (HER-2) ^1^ are the gold standard for predicting patients’ survival and treatment response. Although these prognostic markers are valuable tools in prognosticating tumor burden and progression, additional studies with a longer follow up period indicate that the prognostic value of hormone receptors may not hold true for long-term follow-up studies^2^, and fails to confirm prediction of recurrence and progression^3^. Within the tumor microenvironment, malignant and stromal cells have been shown to play critical roles in secreting multiple factors (such as morphogenic agents) and cytokines, thereby promoting cell growth^4^, angiogenesis^5^, and therapeutic resistance^6^. The increased levels of one or more of these factors (i.e. morphogenic agents or cytokines) in patient’s serum may serve as serum biomarkers for tumor progression, recurrence, therapeutic response and overall survival.

Sonic hedgehog (Shh) is a protein morphogen critical for embryonic development and tissue homeostasis^7^. Tumor cells can produce the Shh ligand that functions either in an autocrine or paracrine manner to promote tumorigenesis and survival of the tumors (reviewed in ref. 8). Studies on many carcinomas, i.e. skin, lungs, breast and prostate, demonstrated ligand-dependent activation of hedgehog (Hh) signaling^9, 10^. Recent evidence suggests that Shh signaling may play critical roles in inducing cancer stem cells (CSCs) thereby accelerating the progression and development of metastasis in solid tumors^11^. Utilizing archived formalin fixed paraffin embedded human breast cancer tumor tissues we have previously shown that higher expression of Shh in triple negative breast cancer (TNBC) patients is correlated with overall patient outcome and survival^12^. Overexpression of Shh in early and late breast cancer stages suggests that modulation of Shh protein might be an early prognostic marker for BC if detectable in serum. Notably, the use of serum Shh as prognostic biomarker from BC patients has not been previously explored. Therefore, we have evaluated blood sera to determine the prognostic significance of serum Shh protein levels from a cohort of BC patients.

Interleukin (IL)-6 is a pleotropic cytokine, constitutively expressed by breast carcinomas. Higher circulating IL-6 levels are correlated with shorter survival in patients with metastatic breast cancer^13^. Response to IL-6 in BC is closely dependent on ER and PR expression implying that hormone sensitive cancer cells are more responsive than hormone insensitive cells. On the other hand, IL-6 has been shown to stimulate estrogen levels in BC^14^. Co-expression and elevation of Shh and IL-6 in serum could be co-regulated, share common mechanisms, and may serve as a biomarker panel. Bieche *et al*.^15^ and Van Laere *et al*.^16^ studied the relationship between Shh and poor outcome in inflammatory breast cancer (IBC) patients, and reported the induction of cancer stem cells (CSCs) by ectopic expression of Shh. Inactivation of Shh signaling reduced the expression of pro-inflammatory cytokines, i.e. TNF-alpha and IL-6, as documented in the gut^17^. Recent publications have identified the importance of IL-6 in the regulation of Shh secretion in the tumor microenvironment. IL-6 is activated and released shortly after tissue injury due to chemotherapy and activated IL-6 results in activation of Shh, which in turn promotes expansion of progenitor populations and leads to the regrowth of tumors^18^. This evidence strongly supports the notion that IL-6 may facilitate the production and distribution of Shh to metastatic sites.

Our previous work was based on immunohistochemical scoring analysis of Shh to identify prognostic values from tumor tissues^12^. Currently, serological prognostic and biomarkers detection have gained momentum in a search for disease onset, disease progression and therapeutic response. Use of serological markers is ideal and less invasive while providing a cost effective test with the potential to provide critical clinical information to monitor tumor burden and progression. We therefore hypothesized that, given the production of Shh and IL-6 in breast tumor microenvironment, serum Shh and IL-6 are abundant and might be detectable in the general circulation, and, if highly abundant in blood sera, would specify poor outcome and prognosis in breast cancer patients. In the present study, we have determined the concentrations of Shh and IL-6 in the blood sera collected from normal volunteers, pre and post chemotherapeutic treated patients representing operable and progressive metastatic BC patients to determine the dual prognostic significance of serum Shh and IL-6.

## Results

### Serum Shh and IL-6 levels significantly differ between healthy subjects and cancer patients

**Figure 1:**
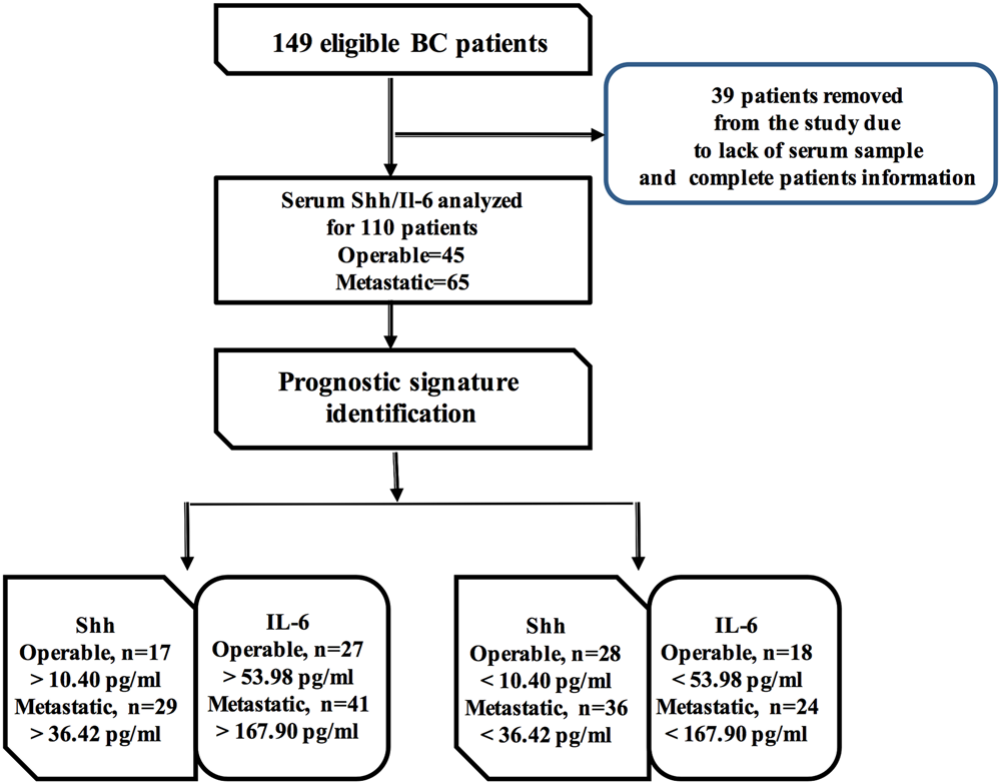
Flow diagram of patients selection and study design. One hundred ten patients were included in the study with serum samples and complete patient’s data, while thirty-nine patients were excluded from the study due to the lack of serum sample and patient’s complete data.

One hundred and ten confirmed BC patients and 30 healthy volunteers were enrolled in this study. Figure 1 shows the patients’ selection and study design criteria. Supplementary Table S1 summarizes the complete patient demographics. At the initial diagnosis, 45 (40.90%) patients presented with untreated early operable tumors, while 65 (59.1%) patients presented with untreated progressive metastatic breast cancer. The median follow-up period for all patients was 36 months.

**Figure 2:**
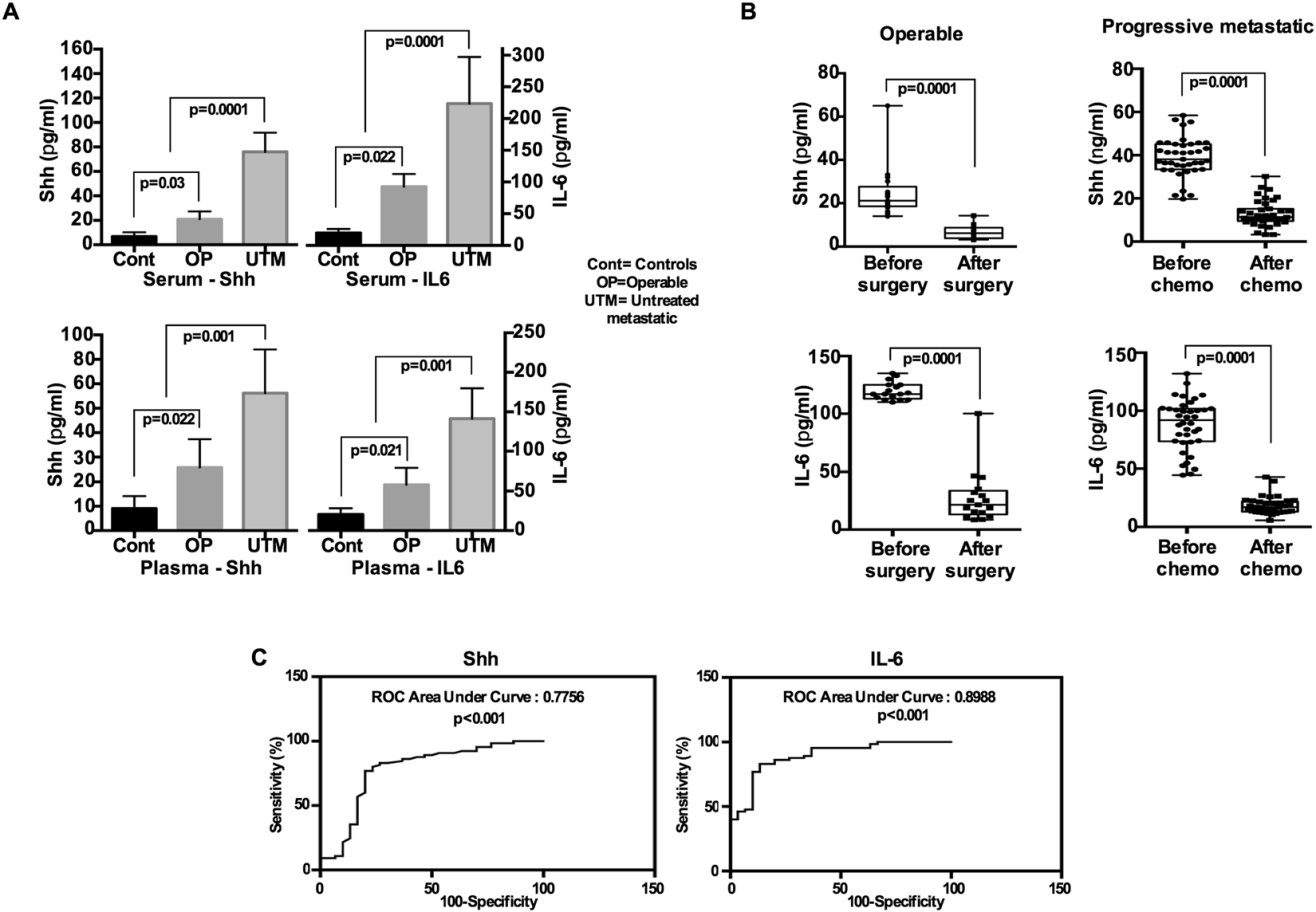
Serum Shh and IL-6 significantly differ between normal and cancer patients. (**A**) Serum and plasma Shh and IL-6 levels from healthy volunteers, early operable and untreated progressive metastatic breast cancer patients. (**B**) Serum Shh and IL-6 levels from early operable patients before and after surgery, as well as before and after chemotherapy from untreated progressive metastatic patients. (**C**) ROC curves of serum Shh and IL-6 as predictors in progressive metastatic breast cancer patients.

The median serum Shh level in the 30 healthy female volunteers was 3.39 pg/mL (95% CI, 2.79–4.03) with a range from 0.5–7.80 pg/mL. Similarly, the median serum IL-6 concentration in this group was 3.14 pg/mL (95% CI, 4.9–17.87) with a range from 4.26–18.42 pg/mL (Fig. 2A). In the 45 patients with early operable BC, the median serum Shh and IL-6 levels was increased to 10.45 pg/mL and 53.98 pg/mL, respectively (Fig. 2A). In the patients with progressive metastatic breast cancer (n = 65) the median serum Shh and IL-6 levels were 37.96 pg/mL and 172.2 pg/mL respectively ranging from 19.10 to 58.4 pg/mL for Shh and 89.47 to 301.4 pg/mL for IL-6 (Fig. 2A). There was a clear significant difference between healthy female volunteers and patients with early operable (p < 0.0001) and progressive metastatic BC patients (p < 0.0001, Fig. 2A).

**Figure 3:**
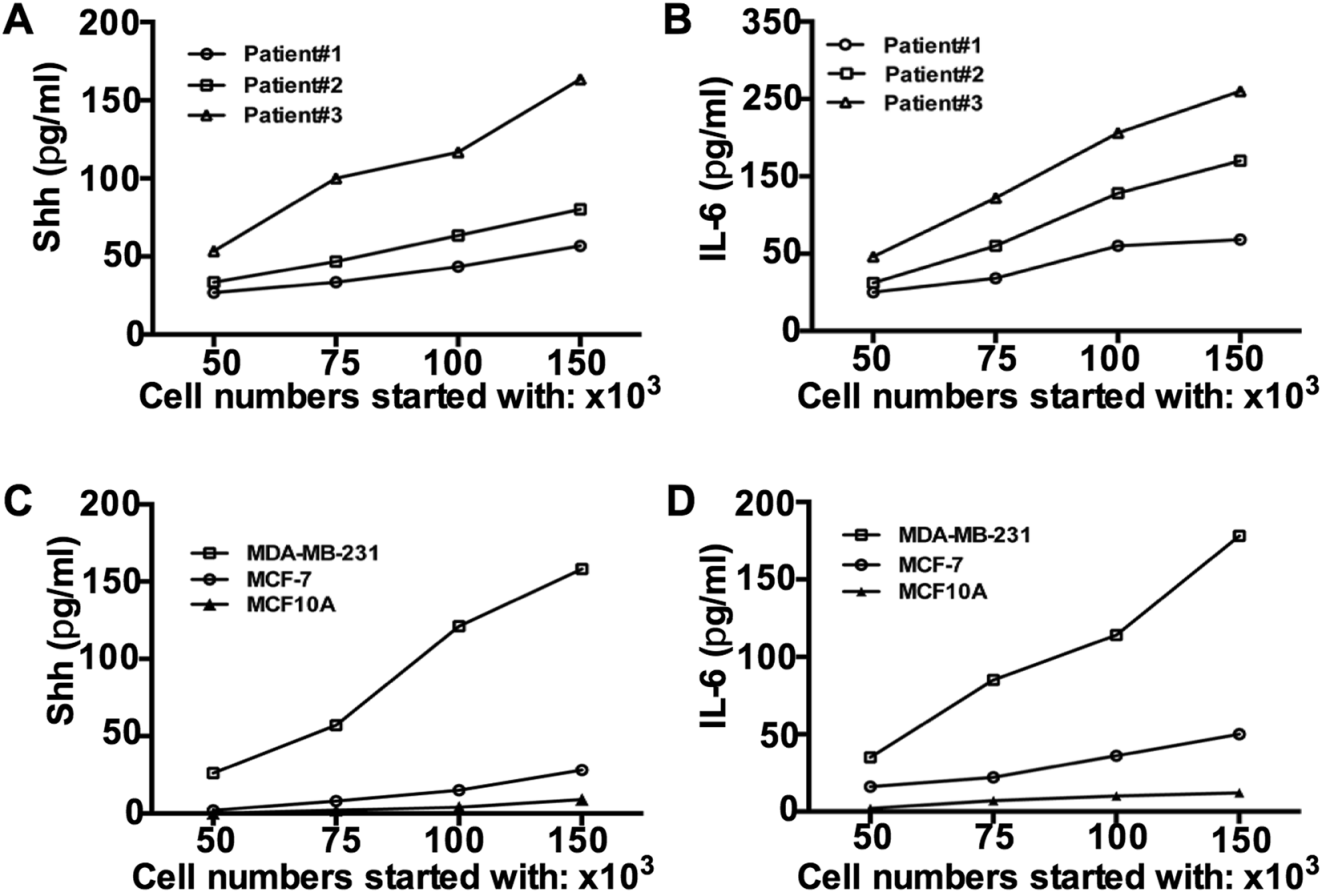
Tumor cell number determines the Shh and IL-6 concentration quantitatively. Freshly collected cells from patient’s tumor samples were cultured for 48-hours in a serum free medium and concentrations of (**A**) Shh and (**B**) IL-6 were measured. Similarly, concentrations of (**C**) Shh and (**D**) IL-6 were measured from MDA-MB-231, MCF-7 and MCF10A cell supernatants.

Plasma Shh and IL-6 levels were also evaluated from the same patients’ samples to identify any correlation between serum and plasma. The median plasma level of Shh in healthy volunteers was 2.59 pg/mL (range 0.40–5.69 pg/mL), while median plasma level of IL-6 was 12.45 pg/mL (range 3.30–15.29 pg/mL, Fig. 3A). Median plasma Shh and IL-6 levels in 45 early operable patients were 9.10 pg/mL (range 3.60–25.71 pg/mL for Shh, Fig. 2A) and 45.80 pg/mL (range 10.70–65.10 pg/mL for IL-6, Fig. 2A). In the progressive metastatic patients group, plasma Shh and IL-6 levels were 27.01 pg/mL (range 10.5–47.60 pg/mL for Shh, Fig. 3A) and 87.60 pg/mL (range 36.24–298.15 pg/mL for IL-6, Fig. 2A), respectively. Thus serum and plasma Shh and IL-6 levels were significantly correlated (r = 0.82, p < 0.001, Supplementary Figure S1).

During the followup period, 29 patients from the early operable patient group had received postoperative systemic therapy and ten patients received chemotherapy. In this longitudinal study, the first blood samples were collected from those patients who underwent tumor resection and both serum Shh and IL-6 levels were analyzed. Indeed, the serum Shh and IL-6 concentrations were significantly reduced after surgery [60 days after surgery] (median 5.40 pg/mL for Shh and 43.98 pg/mL for IL-6), Fig. 3A,B), whereas their serum concentration levels were elevated immediately before [15 days before surgery] surgery in this group (p = 0.0001, Fig. 2A,B).

Similarly, serum Shh and IL-6 levels of thirty-two progressive metastatic BC patients before (21 days before treatment) and after (90 days after treatment) chemotherapeutic treatment were analyzed. The serum Shh and IL-6 levels were elevated immediately before chemotherapy (Fig. 2B). In contrast, both serum Shh and IL-6 levels were significantly decreased after chemotherapy (3.89 pg/mL for Shh and 24.21 pg/mL for IL-6, p = 0.0001, Fig. 2B). Although serum Shh and IL-6 concentrations were lower in the first blood drawn, within three months and after three cycles of chemotherapy serum IL-6 level was elevated slightly in the two consecutive bloods draws while serum Shh remained above the baseline concentration (15.14 pg/mL and 18.47 pg/mL). We next determined the specificity and sensitivity with respect to predictive biomarker potential of serum Shh and IL-6 using the receiver operating characteristics (ROC) curve with an area under curve (AUC) and the values were determined as 0.7756 for Shh [p = 0.001] and 0.8988 for IL-6 [P = 0.001] (Fig. 2C).

### Tumor cell number determines Shh and IL-6 concentrations quantitatively

We preferentially choose Shh and IL-6 because of their overlapping roles in cancer development, progression and possible roles in therapeutic resistance. We then analyzed the Shh and IL-6 concentrations in cell supernatants from freshly harvested tumor cells from patients. Figure 3A,B show the levels of Shh and IL-6 in relation to the numbers of cells initially seeded. Results are shown for three representative patient samples and indicate a variable but definite increase with numbers of cells showing a correlation of Shh and IL-6 for each patient sample. We then asked whether the clinical results could be verified using the well described MCF-10A, MCF-7 and MDA-MB-231 cell lines. MDA-MB-231 cells produced higher amounts of Shh and IL-6 with increasing cell numbers, while the production of Shh and IL-6 in MCF-10A and MCF-7 remained low (Fig. 3C,D). As expected, expressions of Shh and IL-6 in the normal MCF-10A breast epithelial cells were quite low. The better differentiated MCF-7 cell line secreted a modest amount whereas the metastatic MDA-MB-231 cell line secreted levels comparable to patients’ tumors. These results indicate that the number of cells and the malignant phenotype correlate with production of Shh and IL-6 and that secreted Shh and IL-6 could be easily detected in blood serum.

### Cytokines in breast cancer patient’s serum in relation to metastatic status

**Figure 4:**
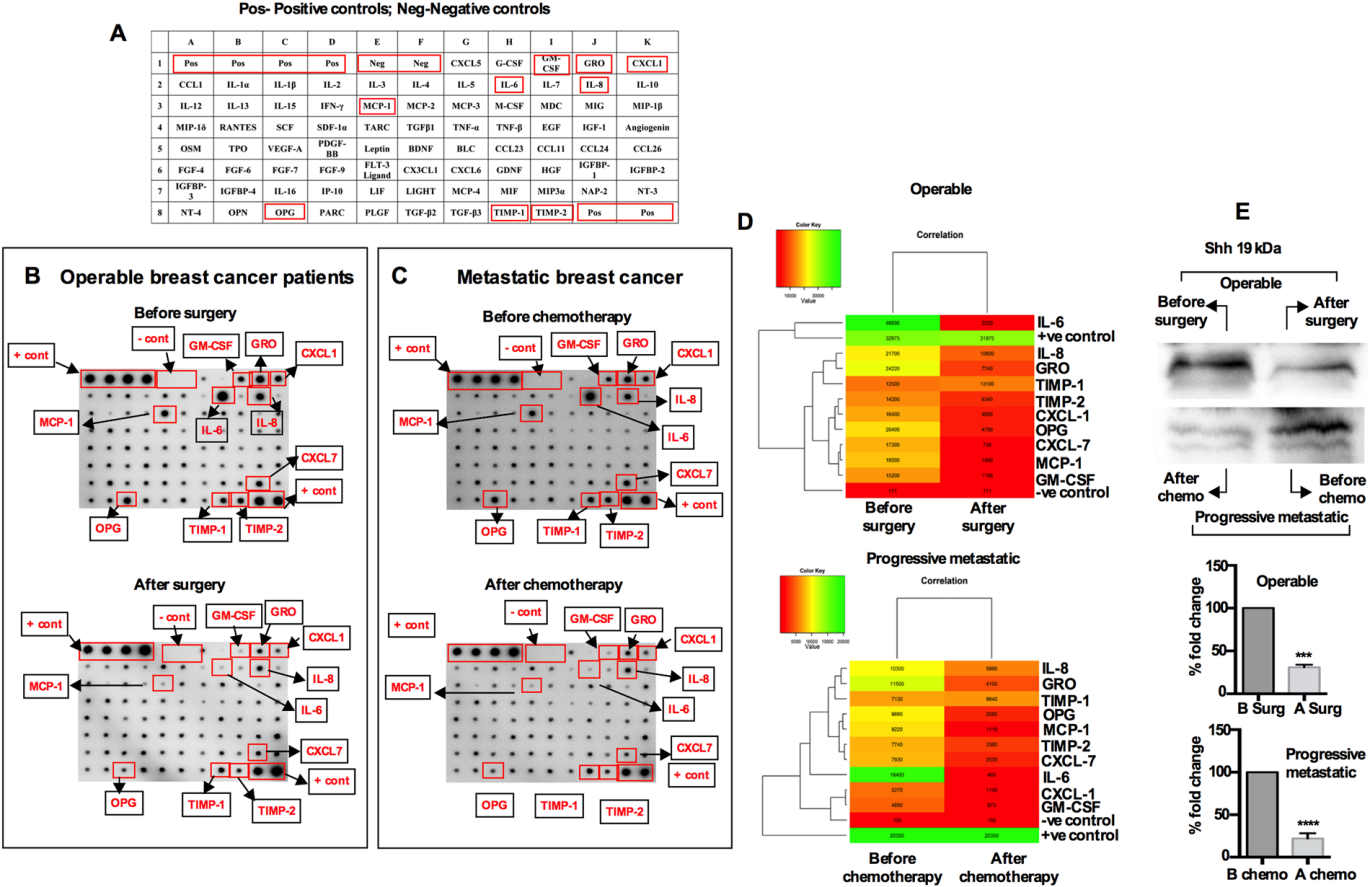
Cytokines in breast cancer patients’ serum. Array analysis of cytokines from breast cancer patient’s serum described in materials and methods. (**A**) Location and map of cytokine antibodies arranged and spotted on the membrane. Representative images of cytokine antibody arrays from (**B**) patients with early operable tumors before and after surgery, and (**C**) from progressive metastatic breast cancer patients before and after chemotherapy. Highlighted rectangles indicate the cytokines elevated. (**D**) Relative expressions of cytokines were determined by densitometer as described in materials and methods and presented as hierarchical clustering (red relatively low and green relatively high). Pos: positive controls, Neg: negative controls. (**E**) Western blot analysis of Shh in serum. Western blot analysis was performed from cohort of early operative (n = 29) and progressive metastatic (n = 20) breast cancer patients and a representative image shown in the figure. Molecular weight of Shh approximately 19 kDa; images shown here have been cropped to show the respective bands. Band densities were quantified with densitometry analysis using ImageJ64 (http://imageJ.nih.gov/ij).

We identified a large panel of cytokines differentially expressed in the breast tumors (see Supplementary Figure S2) from TCGA’s RNA-Seq data portal. As this is based on RNA expression it was important to conduct protein expression profiling using a cytokine antibody array system to identify the key cytokines in serum collected from early operable and progressive metastatic breast cancer patient. Figure 4A shows the position of all cytokines in the membrane. Figure 4B,C show the representative members of the cytokine antibody array data obtained from patients’ serum (n = 110). Both early operable (n = 45) and progressive metastatic (n = 65) breast tumors exhibited strong IL-6 signals that disappeared either after surgery and/or chemotherapy, ﻿respectively, verifying the tumors as the source. The calculated relative densities of all cytokines were determined by densitometric analysis (Fig. 4D; hierarchical heat map). With respect to metastatic potential and ER/PR status, Supplementary Tables S2, S3 and S4 show the cytokines whose expression is associated with ER/PR and metastatic potential. IL-6, IL-8, and TIMP1 are correlated with both metastatic and ER/PR status. The prominence of IL-6 suggests that IL-6 plays a role in BC progression and tumor growth, and therefore based on these results, we restricted our analysis to IL-6 and possible association with Shh^19^. No Shh array kit has yet been developed, and therefore we assessed serum Shh protein levels by Western blot. Serum Shh protein level was elevated 2–3-fold in the serum from pre surgery and chemotherapy patients; the levels sharply declined when patient’s tumor were resected and as well as after chemotherapies (first blood drawn 3 months after surgery and chemotherapy) (Fig. 4E). This finding highlights the possibility that both Shh and IL-6 proteins are retained in serum and transmit coordinated signals to the metastatic sites. To confirm whether IL-6 and Shh participate in promoting metastasis, MDA-MB-231 and MCF-7 cells were exposed to recombinant human IL-6 (15 ng/mL) and Shh (50 ng/mL) proteins and the matrigel coated invasion and migration assay was performed. Both MDA-MB-231 (p < 0.014) and MCF-7 (p < 0.0065) showed an increase in invaded cells through matrigel (Supplementary Figure S3), compared to their controls counterparts. In further support, the invasiveness was significantly attenuated when cells were exposed to anti-IL-6 blocking antibody [15 ng/mL] (p < 0.004 for MDA-MB-231, p < 0.0.002 for MCF-7 cells). To block Shh (p < 0.008 for MDA-MB-231 cell, p < 0.004 for MCF-7 cell) we used the inhibitor cyclopamine [50 uM) (Supplementary Figure S3). When both anti-IL-6 neutralizing antibody (15 ng/mL) and cyclopamine (50 uM) were combined, the attenuation was more pronounced compared to single treatment [p < 0.001 for MDA-MB-231, p < 0.001] (Supplementary Figure S3).

### Sources of Shh and IL-6 in the serum

**Figure 5:**
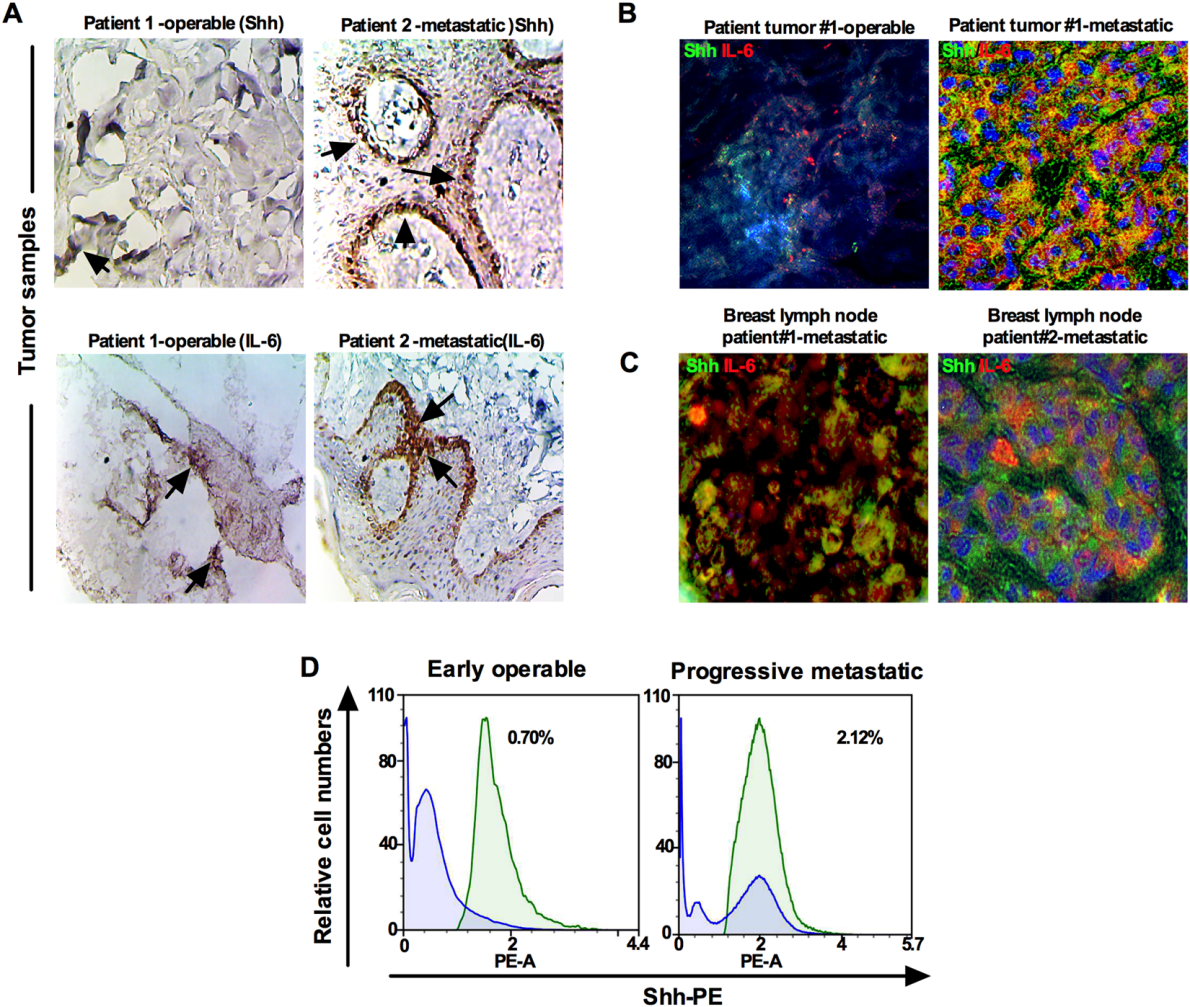
Sources of Shh and IL-6 in the blood serum. (**A**) Immunostaining of Shh and IL-6 in sections from breast cancer patients’ tumors. (**B**) Dual immunofluorescence staining of Shh (green) and IL-6 (red) in tumor samples, and (**C**) Dual immunofluorescence staining of Shh (green) and IL-6 (red) in diagnostic lymph nodes samples. (**D**) Blood cells isolated from early operable and progressive metastatic breast cancer patients were immunostained with Shh-PE-conjugated antibody and analyzed by NovoCyte Flow cytometry. Staining is relative to isotype-matched control. Figure shows that Shh is differentially expressed in patient’s blood cells.

To determine the likely source of Shh/IL-6 production and secretion in serum, we immunostained tissue sections of tumors obtained from early operable and progressive metastatic BC patients as well as lymph nodes (note: no lymph data available from early operable patent). Staining of Shh was predominantly localized in the epithelial compartment with weak staining in stromal fibroblast cells (Fig. 5A,C). Progressive metastatic tumors expressed a high level of Shh versus the early operable group (Fig. 5B). Similarly, epithelial and stromal fibroblast cells were positive for IL-6, with strong expression in the epithelial compartment (Fig. 5A,B). Dual immunostaining of breast tumor and lymph node (from progressive metastatic patient) sections for Shh (green) and IL-6 (red) revealed that both Shh and IL-6 were found within the same epithelial and fibroblast cell compartment (Fig. 5C). Next we determined the existence of Shh + cells in the freshly collected blood from early operable (n = 29) and progressive metastatic patients (n = 32) by using flow cytometry. The percentage of Shh +  cells in progressive metastatic patients was (2.12%) compared to operable tumor patients (n = 29) [0.70%] (Fig. 5D) suggesting a higher degree of circulating tumor cells.

### Prognostic value of serum Shh and IL-6 in the patients with progressive metastatic breast cancer

To analyze prognostic significance of serum Shh and IL-6, univariate and multivariate analyses were applied to a series of 65 patients with progressive metastatic BC patients. Serum Shh and IL-6 were found to be abnormally elevated in progressive metastatic BC patients and Cox-regression model was used to analyze the outcome of EFS and OS. By univariate analysis, the following parameters were considered for EFS and OS determination: tumor burden (<vs≥3.0 cm), tumor progression (low *vs* high), lymph node status (<3 *vs*≥3 or 4+) and median values of serum Shh (<*vs*≥36.42 pg/mL) and IL-6 (<*vs*≥167.90 pg/mL). Median serum Shh (=0.001), IL-6 (p = 0.001) levels, tumor burden (p = 0.020), tumor progression (p-0.031), and lymph node status (p = 0.01) were associated with significant prognostic value and early EFS and shorter survival when elevated. The median survival was shorter (12 months) for patients with serum Shh level above 36.42 pg/mL value by univariate analysis (p < 0.0001) compared to 36 months for other patients that showed lower levels of serum Shh. Survival was significantly shorter (10.5 months) for patients with serum IL-6 level above the 167.90 ng/mL value by univariate analysis.

In addition to univariate analysis, a multivariate analysis of prognostic factors using logistic regression analysis was performed based on the following factors: tumor burden, progression, lymph node positivity and serum Shh and IL-6 levels. Based on the significance values of all five factors described above, tumor burden, progression and lymph node status were eliminated. Using a model in which all these factors were maintained, only serum Shh and IL-6 retained their significance for prognostication (Supplementary Table S5).

### Risk stratification of metastatic breast cancer patient based on serum Shh and IL-6 levels

**Figure 6:**
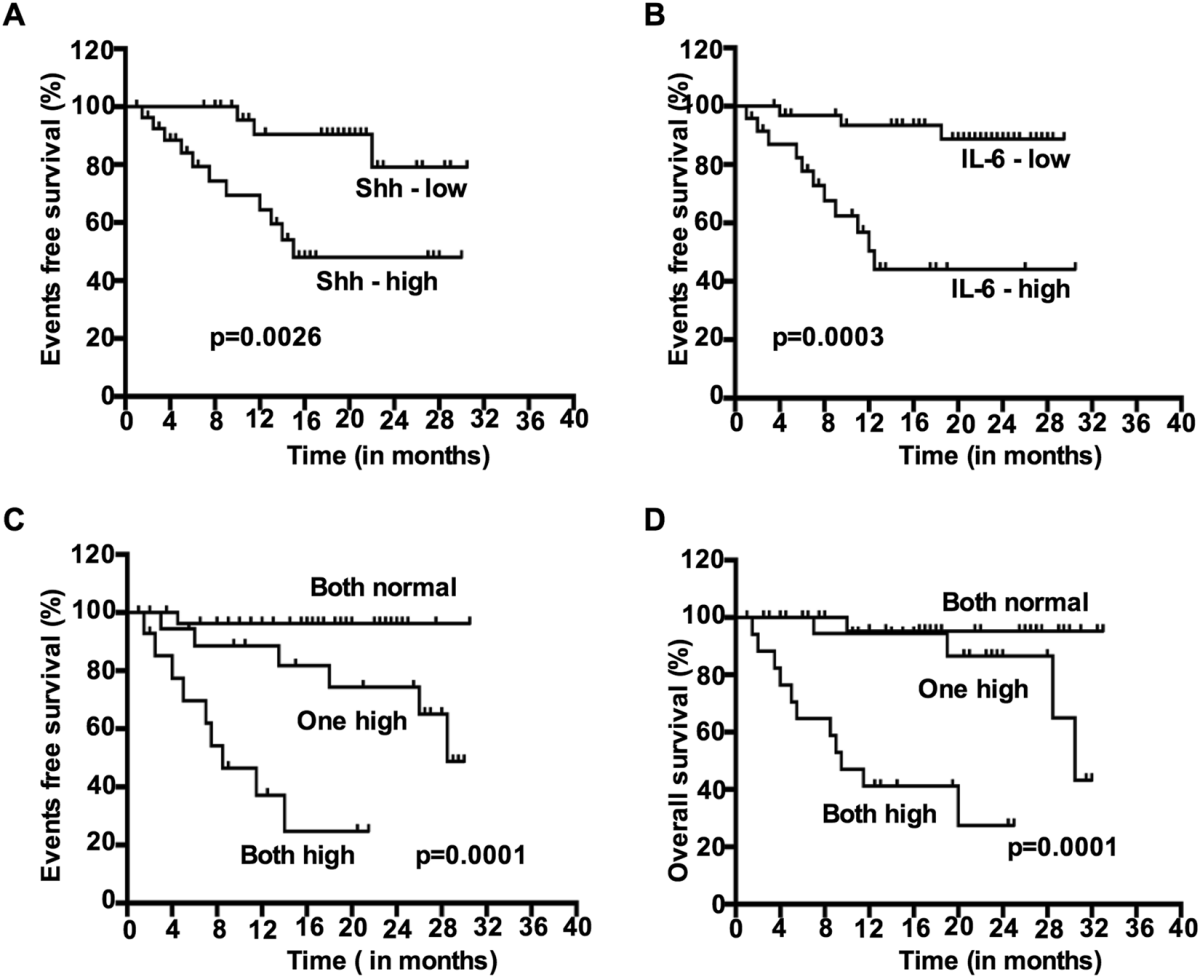
Risk stratification based on EFS and OS correlates with serum Shh and IL-6 levels in metastatic breast cancer. (**A**) Event free survival (EFS) of all progressive metastatic patients by serum Shh levels, (**B**) EFS of all progressive metastatic patients based on serum IL-6 levels, (**C**) EFS of all progressive metastatic patients based on a two-factor model (serum Shh and IL-6). (**D**) Overall survival (OS) of all progressive metastatic patients based on a two-factor model (serum Shh and IL-6). Note worse overall outcome for patients with both Shh and IL-6 at a high level.

Based on the strength of association and cohort size presented by serum Shh and IL-6, we developed a risk stratification model and assessed the EFS and OS. It revealed significant differences in EFS and OS when considering all patients. The serum Shh and IL-6 elevation was associated with EFS at p < 0.001 when both factors were considered individually (Shh: HR = 4.59, p = 0.0026, 95% CI = 1.58–7.36 and IL-6, HR = 5.36.59, p < 0.0003, 95% CI = 2.81–11.24). Both higher levels of serum Shh and IL-6 were independently prognostic for EFS (Fig. 6A,B).

During the follow up period and sample collection time, changes in serum Shh and IL-6 values were observed periodically and based on this observation we categorized patients into three groups: **Group A:** Of 20 patients with normal or lower levels of serum Shh and IL-6, only 3 (15%) had events within 18 months and 2 (10%) had events anytime. **Group B:** Among 16 patients with one factor (serum Shh and/or IL-6) elevated, 6 (37.5%) had events within 14 months, and 5 (31.25%) had events anytime. **Group C:** Among 29 patients with both factors (serum Shh/IL-6) increased, these showed significantly shorter EFS and OS (12 had events within 8 months, 7 had events anytime) compared to women with normal levels of serum Shh/IL-6 or patients who had either one of the factors elevated (HR = 8.36, p = 0.0001, 95% CI 2.81–17.28). Figure 6C (Both normal level, one high and both high, shows the EFS), and Fig. 6D (OS of all patients by the two factor, serum Shh and IL-6, elevation model) are given.

### Bone metastasis and elevated level of serum Shh and IL-6 in progressive metastatic breast cancer patients

**Figure 7:**
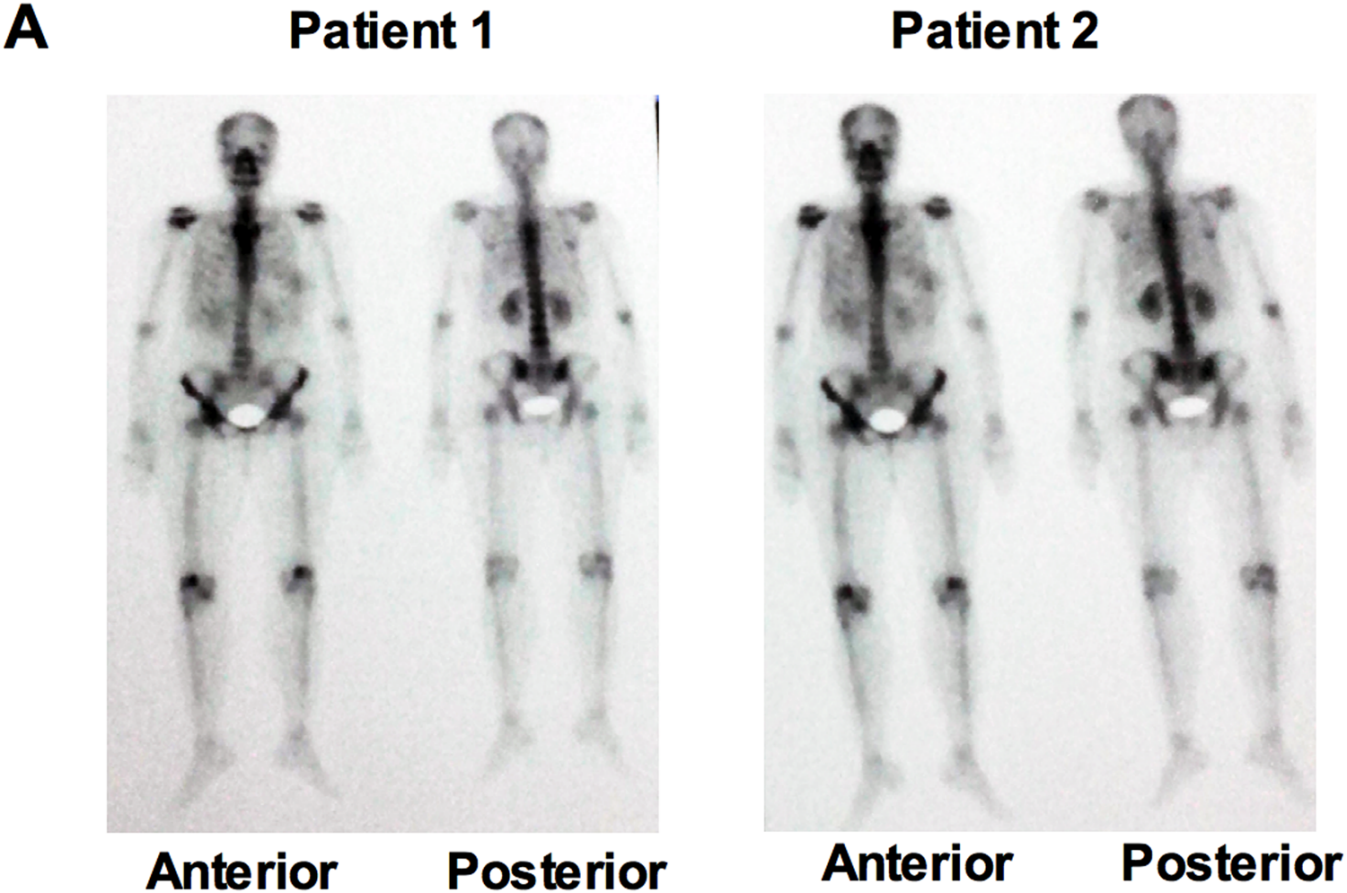
Bone metastasis and elevated levels of Shh and IL-6 in progressive metastatic breast cancer patients. (**A**) Scintigraphic imaging of whole body scan for the detection of bone metastasis from advanced stage progressive metastatic breast cancer patients showing anterior and posterior view of both shoulder and knee joints.

Bone scintigraphy is commonly used as a primary imaging procedure for detecting and monitoring real time progression of bone metastasis. A whole body image was acquired (during the followup period as requested by the care provider) of the anterior and posterior aspects within 3 hours after intravenous injection of 20 mCi of 99mTechnetiumhydromethlene diphosphate (99mTc HDP). In four patients studied, whole body scintigraphy detected abnormalities, suggesting bone metastasis involvement and/or osteoarthritic changes. Figure 7A shows the diffusely increased radiotracer uptake at both shoulders and knee joints. There is a strong correlation between elevated serum Shh and IL-6 and increased radiotracer uptake and bone metastasis in the patients. These patients showed clinically recurrent disease during follow up period and two patients died within 6 months of initial diagnosis. Although we were unable to collect bone marrow aspirates to confirm the bone metastasis using specific marker analysis, these tentative findings suggest Shh and IL-6 involvement in bone metastasis.

## Discussion

Current breast cancer therapy generally involves some combination of surgery, local radiation therapy, applying of systemic chemotherapy and targeted therapy, resulting in improved patient’s OS. However, over-diagnosis can incur a financial burden for both the health care provider and patients. On the other hand, overtreatment can cause severe side effects for the patients. Recent approaches for prognosticating tumor burden and prognosis based on stratifying patients risk group is of special interest. Moreover, the predictive parameters for predicting therapeutic response and efficacy is in high demand to avoid over-diagnosis and overtreatment. Due to cellular and molecular heterogeneity of breast cancer, patients with same stage and grade of disease may respond very differently to therapy implying that traditional parameters fail to confirm prognosis and prediction. Recent advancements in gene expression profiling hold the promise for providing new avenues for prognosticating and predicting breast cancer outcomes^20^. Since, gene expression studies mainly rely on the informative value of the whole tissue specimen, therefore gene expression based prognostication and prediction has several limitations. One such critical limitation is that the gene expression based prognostication approach does not allow uninterrupted patient monitoring and assessment during and/or after treatment and surgery. To overcome this limitation, non-invasive and inexpensive serum based analysis of tumor markers is particularly valuable for monitoring disease progression and treatment efficacy. Evidence suggests that tissue leaked proteins^21^ provide useful clinical information which serve as indicators in a variety of human diseases. We investigated whether information stored in serum could provide critical outcome and assist in breast cancer management. Our study has demonstrated that the highest levels of both serum Shh and IL-6 correlated with a higher risk of early relapse compared with that in patients with negative or lower level. This study further demonstrated that serum Shh and IL-6 levels (both levels are higher than baseline levels) remained a significant predictor of EFS and OS when adjusted for other known factors i.e. tumor grade, lymph node status and progression. These results indicate that measurement of serum Shh and IL-6 may be clinically useful, constituting additional biomarkers to be added to the growing body of evidence for risk stratification and prognosis in BC patients.

Our assumption was that elevated levels of serum Shh and IL-6 would portend a greater risk of metastatic breast cancer, the assumption being credible because Shh and IL-6 are potent promoters of growth, survival, and migration of breast cancer cells^22, 23^. We have now demonstrated the prognostic impact of serum Shh and IL-6 in patients with progressive metastatic BC. The strength of this work lies in the fact that patients were enrolled in this prospective study at the time of diagnosis and all patients were untreated at the time of sample collection implying that serum Shh/IL-6 mirrors tumor progression. Patients who underwent several cycles of chemotherapy during our followup period observed a sharp decline in serum Shh/IL-6 levels in the first three months, however, serum Shh levels then resumed to be elevated again in the subsequent blood drawn six months after surgery and chemotherapeutic treatment, however this was not statistically significant. This was likely due to a developing resistance to therapy since increased serum Shh/IL-6 might reflect an association with resumed tumor growth based on their association with induction of the cancer stem like cells.

Previous studies including our own and others using FFPF tumor tissues have shown that Shh is predominantly expressed in a subset of tumors i.e. TNBC tumor samples^12, 24^. Histological diagnosis of breast cancer occasionally may be difficult due to the variety of cytomorphological variations. Therefore, assessing serum biomarkers for metabolites, adhesion molecules, angiogenesis factors, and cytokines are under current investigation in many cancers^25^. In patients with BC, the prognostic relevance of serum Shh has not been previously reported. El-Zaatari *et al*.^26^ reported reduced plasma Shh levels in pancreatic cancer patients. From this study, it would appear that Shh is secreted from tissues and organs into the circulation but its activity could be blocked by plasma protein. Our analysis of serum Shh revealed a significantly lower amount in healthy volunteers compared to early operable, with the highest serum Shh levels in the progressive metastatic BC patients group. Both serum and plasma levels were equivocal in our study. These findings suggest that progressive metastatic BC patients tend to have elevated serum Shh, but then this gradually decreases once patients’ tumors have been resected or after chemotherapy. Overall our study revealed two important observations about serum Shh: (a) serum Shh levels correlate with tumor burden and progression, (b) serum Shh levels tend to decrease upon surgery or treatment. Our findings suggest that serum Shh expression at the time of diagnosis of metastasis is an early indicator of overall unfavorable outcome and EFS in BC patients.

The individual prognostic value of IL-6 has previously been demonstrated in other malignancies^3, 27–31^. In the series of sixty-five untreated progressive metastatic BC patients, 81% (53 out of 65) showed serum IL-6 level above the detection limit with significantly higher in those patients with a greater metastatic tendency. These results are in agreement with previously reported results in BC patients. Patients with normal or low levels of serum Shh and IL-6 have better favorable survival and EFS, compared with patients whose serum Shh and IL-6 levels continue to increase. These increases may be associated with early relapse. A worse prognosis was associated with higher levels compared with whose serum Shh and IL-6 levels fell below the limit in normal volunteers. This supports the contention that serum Shh and IL-6 levels when higher than normal are associated with early relapse, EFS, and poor OS.

Several earlier reports indicate that Shh and IL-6 are produced by epithelial cells and stromal fibroblast cells within the tumor microenvironment and both epithelia and stroma respond to the signal via paracrine or autocrine processes^32, 33^. Although our study is not the first to report the source of Shh and IL-6, individually, in agreement with others our findings indicate that epithelial cells express a higher level of Shh and IL-6 than stromal fibroblasts, suggesting that Shh and IL-6 produced by both epithelial cells and stromal cells are released into blood serum that can lead to enhanced metastatic progression of tumor cells. Additionally, dual immunofluorescence staining for both Shh and IL-6 of diagnostic lymph node specimens further confirmed that the primary tumor microenvironment is not the only source of serum Shh and IL-6. Our results suggest that elevated levels of serum Shh and IL-6 most likely also originate from lymph node metastases, potentially, and overall are likely accountable for the increased tumor burden, and life-threatening progression and metastasis.

Our observation on serum Shh/IL-6 revealed disease progression in those patients under therapy. This is possibly due to the fact that both Shh and IL-6 are involved in mediating drug resistance. In patients with recurrent BC, IL-6 associated anthracycline resistance has been reported^34^. Furthermore, endocrine therapy with medroxy progesterone acetate (MPA) showed similar association with response to treatment^35^. On the other hand, Shh inhibitors could potentially inhibit the incidence, multiplicity and expression of IL-6 described recently^19^. This raises the possibilities of utilizing Shh inhibitors to overcome IL-6 induced tumor progression. From the standpoint of treatment, both Shh and IL-6 signaling networks have been targeted with therapeutic regimens in several cancers conducted in preclinical and clinical trials^36, 37^. Our results also provide evidence that patients with metastatic and ER/PR status is correlated with serum Shh and IL-6 levels as these factors have direct implications for developing therapies^38^. Although this study did not categorize/emphasize molecular subtypes (ER/PR/HER2 status), nervertheless we observed serum Shh and IL-6 levels were detected mostly in ER/PR-negative patients, commonly known as triple negative breast cancer (TNBC), implying that serum Shh and IL-6 could be additionally used to monitor predictive therapeutic response. Of note, recent evidence suggests that Shh and IL-6 play major roles in initiation, maintenance and drug resistance in breast cancer^39^. We suggest that future work should place emphasis on how serum levels of Shh and IL-6 could be utilized as prognostic biomarkers in the light of the ER/PR/HER2 prism. Taken altogether, we suggest an approach to measure serum Shh and IL-6 levels would be clinically useful. The rationale for using these prognostic markers for risk assessment and therapeutic response requires further strenthening.

Bone is a preferred metastatic site for breast cancer cells and interacts with cells within the bone microenvironment. Through the vertebral venous system cancer cells are transported and come into contact with the axial skeleton, including ribs, spine, pelvis and humerus and femurs^40^. Once cancer cells have migrated to bone, various growth factors, i.e. VEGF and cytokines including IL-6, are secreted and induce osteolytic activity. However, we are unaware of any study that has addressed the question whether elevated serum Shh and IL-6 levels correlates with bone metastasis and osteoarthritis. We are the first to show that there is a significant correlation between bone and elevated levels of serum Shh and IL-6. The limiting factor for this observation is that we were unable to analyze bone marrow aspirates, however, imaging of whole body by scintigraphy revealed a higher uptake of radiotracer in the shoulder and knee joints supporting our results that an elevated level of serum Shh together with IL-6 could play a critical role in BC metastasis to bone.

In conclusion, we have addressed a clinically relevant problem with an observation of noteworthy association between elevated serum levels of both Shh and IL-6 and clinical outcome of BC patients. These two factors taken together appear to be strongly predictive of a patient’s response. This study investigated serum from a small cohort of one hundred and ten BC patients and thirty healthy volunteers. Further studies utilizing a larger cohort and longer followup period could find a stronger relationship between breast cancer progression and Shh and IL-6 for testing their prognostic values and predictive therapeutic responses for these two biomarkers in combination with other biomarkers such as CA15.3^41^.

## Materials and Methods

### Samples and study design

After obtaining written informed consent (Chittagong Medical College and Hospital), patients diagnosed with BC were prospectively recruited for this study at the University of Chittagong and Chittagong Medical College Hospital during hospital and clinic visits. This study protocol was approved by Chittagong Medical College and Hospital and University of Chittagong Institutional Review Board (IRB). All methods described in this study were performed in accordance with the relevant national (Bangladesh Medical Research Council, Bangladesh, BMRC) guidelines and regulations. This study was designed to investigate three groups of recruited patient populations 1) A control group with a total of 30 healthy female volunteers. 2a) A first patient group (n = 45) with confirmed breast cancer which at the time of diagnosis were determined as early operable disease. 2b) A second group with untreated progressive metastatic breast cancer (n = 65). All patients in groups 2a and 2b consented to have blood testing. Blood samples were collected from the patients and bloods were centrifuged to separate serum and plasma and stored at −80 °C. All enrolled patients were monitored systematically for event-free and overall survival (EFS and OS, respectively). Blood was drawn a second time at the first followup (1–3 months later) and every 6-months over a 3-year period.

### RNA-Seq data source and data analysis

RNA-Seq data (Illumina HiSeq 2000 RNA sequencing platform) on 1097 breast invasive carcinoma (BRCA) tumor samples were downloaded from The Cancer Genome Atlas (TCGA) for this study and processed as described previously^12^.

### Cell cultures

Freshly resected tumor tissues were minced and incubated with Dispase II (5 mg/ml) for 18 hours at 37 °C on a rotary shaker according to manufacturer’s instruction (Cat no: 04942078001; Roche). Briefly, the supernatants were discarded after centrifugation and pelleted cells were collected and cultured in RPMI-1640 medium supplemented with 10% FBS and 1% antibiotic. Once cells reached 90% confluency, cells were dissociated in 0.25% Trypsin/EDTA (cat no: 25–200–114; Gibco, Canada) and re-cultured at required numbers of cells (50,000, 75000, 100000 and 150,000 cells). After 48-hours, culture medium was replaced with serum free medium. After 48 hours, collected serum free media were subjected to ELISA assay. MDA-MB-231, MCF-7 and MCF10A cells were obtained from American Type Cell Cultures (ATCC) and cultured as recommended. As above, the required numbers of cells were plated and after 48 hours the medium was replaced with serum free medium and incubated for another 48 hours, and finally cell supernatants were collected and subjected to ELISA assay.

### Measurement of serum Shh and serum cytokine IL-6

Serum Shh and IL-6 concentrations were measured from pre and post treatment blood draws, using human Shh (cat no: ELH-ShhN) and IL-6 (cat no: ELH-IL-6-1) specific ELISA Kits (RayBiotech) following manufacturers protocols. Briefly, samples of diluted 100 μl of serum and/or standards were incubated on ELISA plates at 4 °C overnight with gentle shaking. The solution was discarded, washed with 1x wash buffer (WB). Biotinylated antibody was added to each well and incubated for 1 hour at RT with shaking. Washed wells with WB, and 100 μl/well of streptavidin added and incubated at RT for 45 minutes. After washing, One Step Substrate Reagent TMB (3,3′,5,5′-tetramethylbenzidine) was added to each well and incubated for 30 minutes at RT. Added 50 μl of stop solution and read the plate in a BioRad Microplate Reader at 450 nm. The minor optical imperfections in the ELISA plate were considered and readings optimized by subtracting the values at 570 nm. The mean absorbance was calculated from a set of duplicate standards, controls, and samples by subtracting absorbance values from blank. Data were plotted as the fold-change in Shh and IL-6 levels over the controls.

### Cytokine array

Cytokine levels from sera were assayed using RayBio C-Series Human Cytokine Antibody Array C5 (cat no: AAH-CYT-5; RayBiotech, Norcross, GA) following manufacturer’s protocols. Briefly, membranes were placed into incubation tray containing blocking buffer and incubated for 30 minutes at RT. After blocking, diluted (1:3) serum were added to the membrane and incubated at 4 °C overnight, followed by washing twice with buffer, and incubated with Biotinylated Antibody Cocktail overnight at 4 °C. After washes, membranes were incubated with HRP—Streptavidin Concentrate for 2 hours at RT, washed twice, and placed into the detection buffer for 2 minutes. Signals were detected by ImageQuant Imaging System. Relative cytokine levels were quantified by densitometric analysis using ImageJ (NIH, Bethesda, MD).

### Invasion assay

Cell invasion was performed using BD Biocoat chambers (cat # 354578, BD Bioscience, USA) containing filter with 8.0 μm pores coated with 15.0 μg of Matrigel (BD Bioscience, USA). MDA-MD-231 and MCF-7 cells were cultured in serum free media and treated with 15 ng/mL of recombinant IL-6 and Shh (50 ng/mL) and incubated for 12 hrs at 37 °C in humidified incubator. For inhibition of IL-6 and Shh mediated invasion, anti-IL-6 antibody (15 ng/mM) and cyclopamine (10μM) were added and incubated for overnight. Cells were trypsinized and seeded 20,000 cells/well in Matrigel coated upper chamber and complete medium was placed in the lower chambers as chemoattractant. Invasion and migration were scored after 24 hrs at 37 °C in humidified 5% CO_2_ incubator. Non-invading and migrating cells were removed from the upper surface and invaded cells on the lower surface were fixed with 1% crystal violet (Fischer Scientific) in 20% methanol. Stained cells were counted from at least five random fields at 40x magnification.

### Western blot

Western blotting was performed as described previously^42^. Primary antibodies used were goat polyclonal to Shh (N-19) (cat# sc-1194, Santa Cruz, USA) and goat polyclonal to IL-6 (M-19, cat# sc-1265, Santa Cruz, USA).

### Immunostaining

Immunohistochemical and Immunofluorescence staining were performed as described previously^43^. The primary antibodies used were those as described for Western blotting.

### Flow cytometry

Flow cytometry was used to analyze the Shh-positive cells in patients’ blood samples. To analyze the serum Shh expression in the blood cells, cells were immunolabeled with a single Shh-PE-conjugated mouse monoclonal antibody (cat no: IC4641P; R & D System) for 1 hour at RT. Cells were washed with WB and were analyzed by flow cytometry (NovoCyte, USA). Isotype matched controls was used as negative control.

### Statistical analysis

The primary analysis was performed to determine the expression profile of selected cytokines and Shh from TCGA archived RNA-Seq RPKM values from breast cancer patients. “R” Statistical software (Version 3.3.0) was used for making heatmap plots and analyzing data. Prism Graph Pad statistical software (San Jose, USA) was used to determine the relationship between Shh and IL-6 levels and both EFS and/or OS. EFS was determined as time from initial diagnosis to disease progression, retreatment or death due to cancer related and unrelated cause. Cox proportional hazards regression models were used to determine the association of Shh and IL-6. Kaplan-Meier survival curves were used to represent data graphically to show the association of Shh and IL-6 with outcome.

## Electronic supplementary material

Supplementary data 1
