# Supplementary material for: Serum sonic hedgehog (SHH) and interleukin-(IL-6) as dual prognostic biomarkers in progressive metastatic breast cancer
Source: Sci Rep. 2017 May 11;7:1796. doi: 10.1038/s41598-017-01268-4 (PMC5431756; doi:10.1038/s41598-017-01268-4)

**Supplementary data:**

**Serum sonic hedgehog (SHH) and interleukin-(IL-6) as dual prognostic biomarkers in progressive metastatic breast cancer**

Noman AS^1^, Uddin M^2^, Chowdhury AA^3^, Nayeem MJ^1^, Raihan Z^1^, Rashid MI^1^, Azad AK^4^, Rahman ML^1^, Barua D^1^, Sultana A^1^, Sathi AS^1^, Ferdous J^1^, Parag RR^1^, Rahman SM^1^, Mahmud MR^1^, Jerin CS^1^, Jahan N^1^, Siddiqua A^1^, Ara T, Sabur EB^1^, Alam SS^5^, Baidya S^1^, Akther S^1^, Rahman MZ^6^, Banu T^7^, Murugan AK^10^, Sabri S, Islam SMS^8^, Karakas B^9^, Aboussekhra, A^9^, Yeger H^10^, Farhat W^10^**,** Islam SS^9; 10, *^

Supplementary Table S1: Clinical characteristics 110 breast cancer patient.

| **Demographics** | **Breast cancer patients** | | | | | |
| --- | --- | --- | --- | --- | --- | --- |
|  | **Progressive metastatic breast cancer patients** |  |  | **Operable patients** |  |  |
| Age | 56 |  |  | 52 |  |  |
| Number of patients | 65 |  |  | 45 |  |  |
| Grade I/II/III | 17/23/25 |  |  | 27/11/7 |  |  |
| Blood collection before surgery (in day) | 15(1.1)^#^ |  |  | 15(1.0)^#^ |  |  |
| Blood collection after surgery (in day) | 45(1.1)^#^ |  |  | 45(1.1)^#^ |  |  |
| ER+/ER- | 35/21^*^ |  |  | 37/12^*^ |  |  |
| PR+/PR- | 37/19^*^ |  |  | 33/17^*^ |  |  |
| Tumor size (cm) | 5.9 (1.54)^#^ |  |  | 2.80 (0.98)^#^ |  |  |
| Lymph node+/Lymph node- | 24/30^*^ |  |  | 9/21^*^ |  |  |
| Chemotherapy/No-chemotherapy | 32/17^*^ |  |  | 20/24^*^ |  |  |
| Ductal/Lobular | 39/17 |  |  | 31/21^*^ |  |  |
| Adjuvant therapy (Hormonal/Tamoxifen) | 9 |  |  | 22 |  |  |
| Adjuvant therapy (aromatase inhibitor) | n/a |  |  | n/a |  |  |

Supplementary Table S2: Expression of cytokines with ER status

| **Cytokines** | **ER+** | **ER- p-values** |
| --- | --- | --- |
| IL-6 | 🡻  | 🡹  0.021 |
| IL-8 | 🡻 | 🡹  0.014 |
| TIMP1 | 🡻 | 🡹  0.031 |

Supplementary Table S3: Expression of cytokines with PR status

| **Cytokines** | **PR+** | **PR- p-values** |
| --- | --- | --- |
| IL-6 | 🡻 | 🡹 0.034 |
| IL-8 | 🡻 | 🡹 0.014 |
| TIMP1 | 🡻 | 🡹 0.034 |

Supplementary Table S4: Expression of cytokines with TNBC status

| **Cytokines** | **TNBC** | **Non-TNBC p-values** |
| --- | --- | --- |
| IL-6 | 🡹 | 🡻 0.021 |
| IL-8 | 🡹 | 🡹 0.011 |

Supplementary Table S5: Results of multivariate analysis: significance factors and correlation

| **Factors** | **HR^*^** | **95% CI^#^** | **P values** |
| --- | --- | --- | --- |
| Tumor burden (< *vs* ≥3.0cm) | 1.74 | 1.10-2.59 | 0.020 |
| Tumor progression (low *vs* high) | 2.10 | 1.45-3.57 | 0.011 |
| Lymph node status (<3 *vs* ≥3 or 4+ | 2.10 | 1.03-3.24 | 0.010 |
| Shh (< *vs* ≥36.42 pg/mL) | 2.58 | 1.71-3.54 | 0.001 |
| IL-6 (< *vs* ≥167.90 pg/mL | 3.01 | 1.87-4.27 | 0.001 |

**Supplementary Figure Legend**

**Supplementary Figure S1: 1A and 1B)** Correlation of serum Shh and plasma Shh **1C and 1D)** serum Shh and plasma IL-6 in early operable and progressive metastatic breast cancer patients

**Supplementary Figure S2: 2A)** Heat map representation of 1097 breast cancer patient’s RNA-Seq transcript analysis from TCGAs repository. Data were downloaded from TCGAs repository and analyzed the expression pattern for cytokines and hedgehog pathway family genes.

**Supplementary Figure S3:** Numbers of invaded cells of 3A) MCF-7, and 3B) MDA-MB-231 cells after incubation with recombinant IL-6 (15ng/mL) and anti-IL-6 neutralizing antibody (0.25ng/mL). 3C) MCF-7, and 3D) MDA-MB-231 cells. Cells were treated with recombinant Shh (50ng/mL) and cyclopamine (10uM) and invasive capacity was assessed. 3E) MCF-7 and 3F) MDA-MB-231 cells. Cells were treated with combination of recombinant IL-6 and Shh and the invasion potential were inhibited with anti-IL-6 neutralizing antibody and cyclopamine.

**
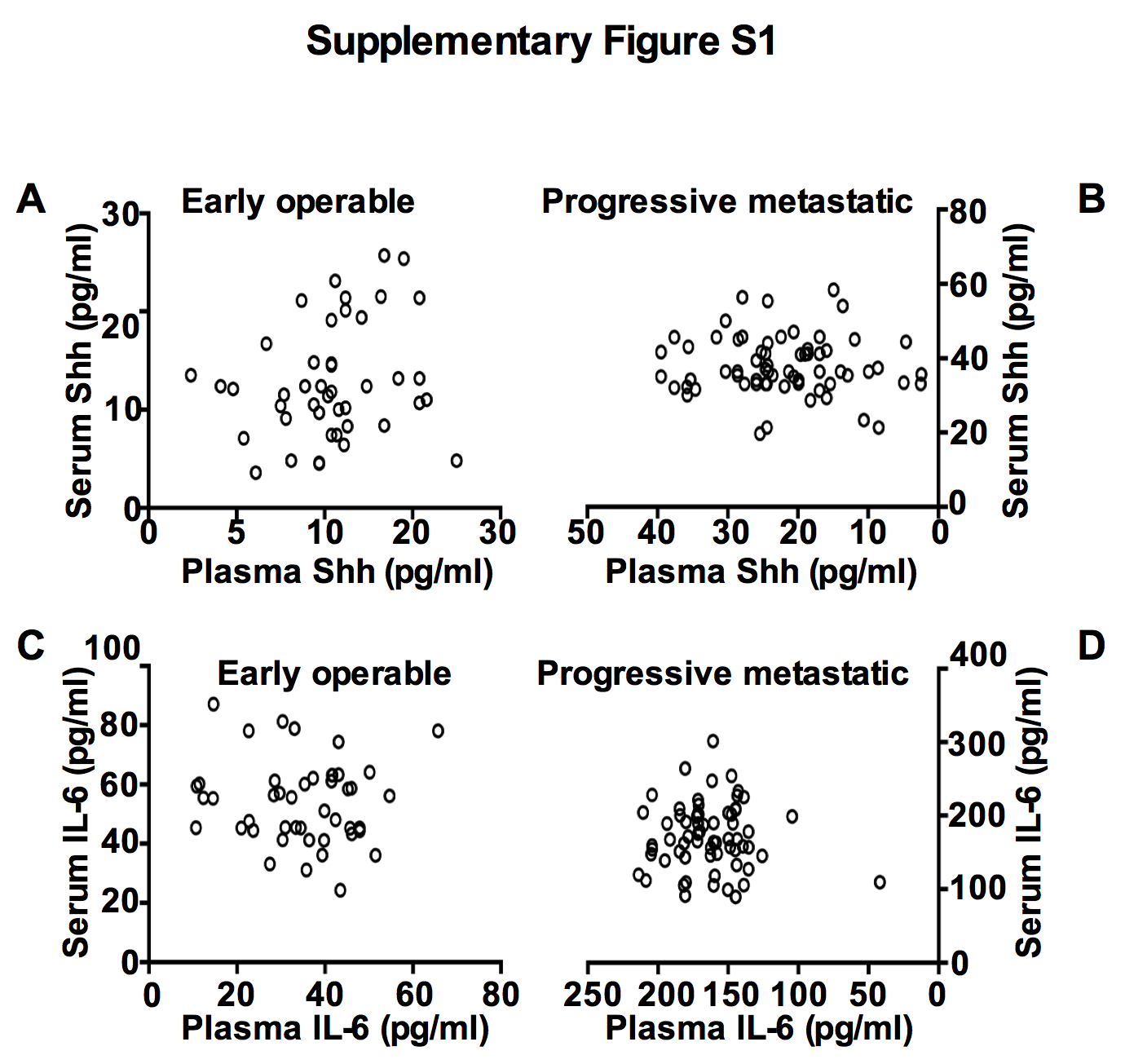
**


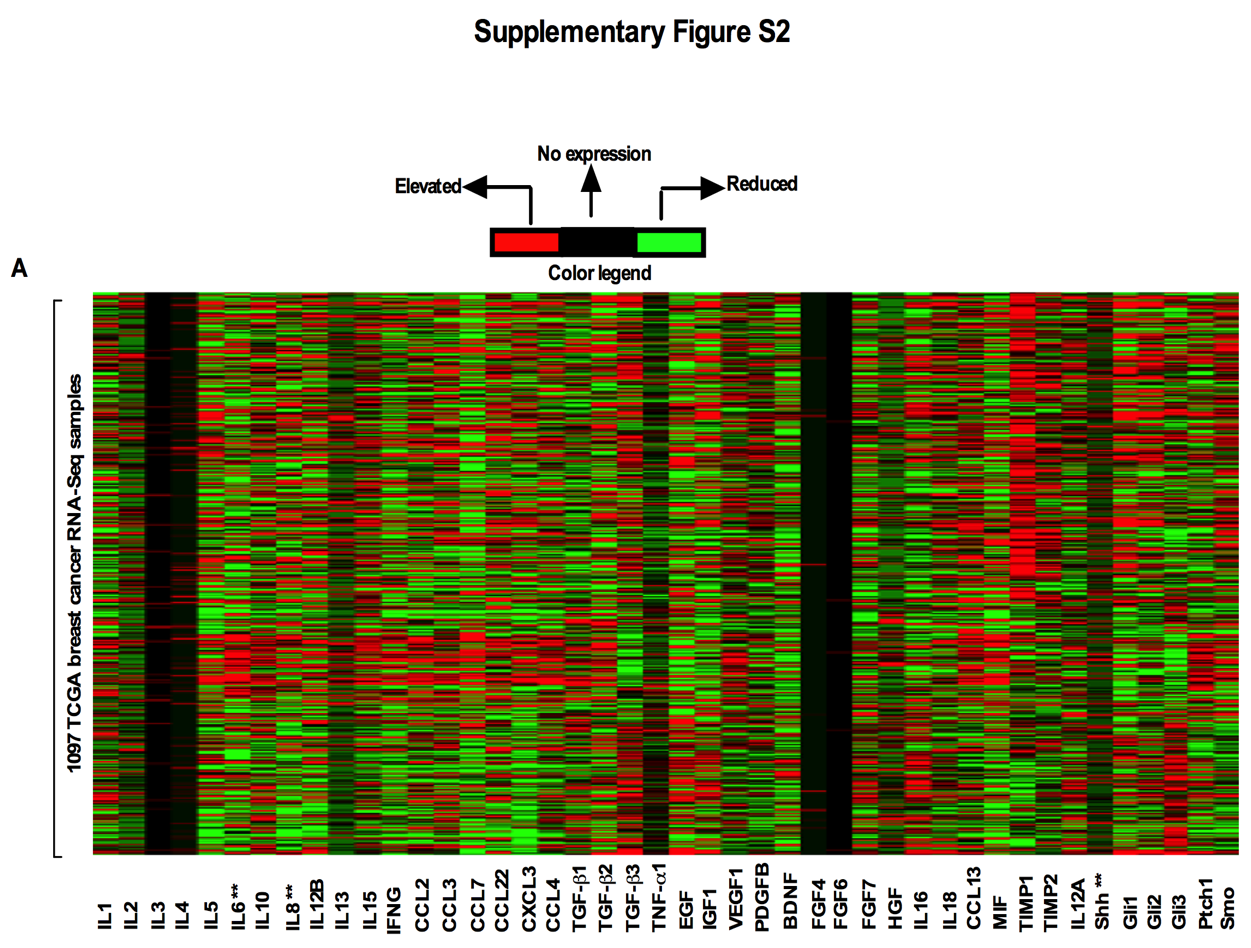


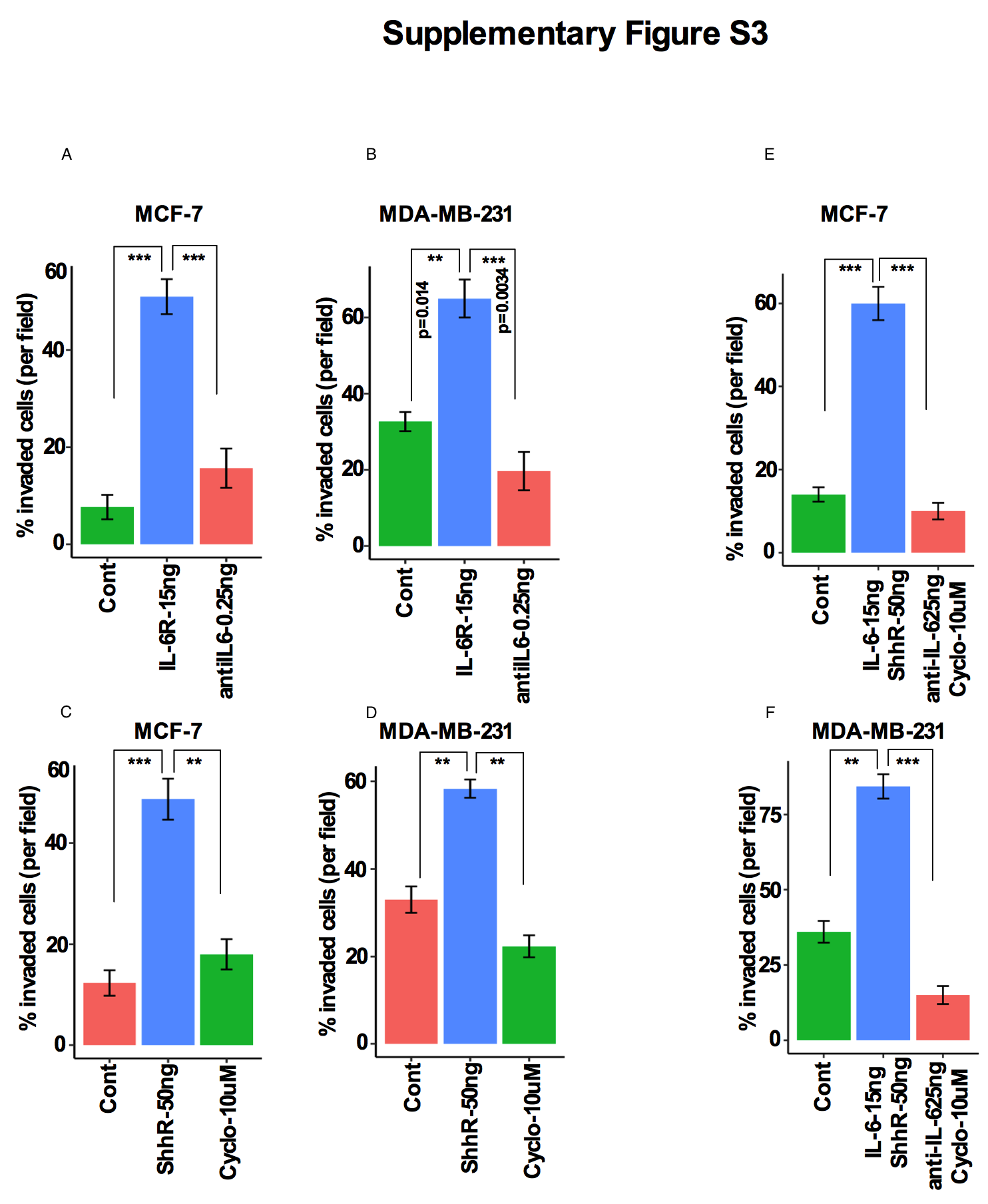

Supplement: Supplementary file 1 — Supplementary data 1 [file 41598_2017_1268_MOESM1_ESM.doc]
